# A Case Report of Recurrent Hypokalemic Quadriparesis in the Setting of Distal Renal Tubular Acidosis Preceding Typical Sicca Symptoms in Primary Sjögren’s Syndrome

**DOI:** 10.7759/cureus.70185

**Published:** 2024-09-25

**Authors:** Yarrabathina Laxmi supriya, Ridhinayani Nalam, Sai Pavan Lagishetty, Pravalika Meka, Greeshma Rangari

**Affiliations:** 1 Medicine, Gandhi Medical College, Secunderabad, IND; 2 Internal Medicine, Kaloji Narayana Rao University of Health Sciences, Hyderabad, IND; 3 Internal Medicine, Government General Hospital, Nizamabad, IND; 4 Internal Medicine, Government Medical College, Nizamabad, IND

**Keywords:** case report, distal rta, primary sjögren’s syndrome, recurrent hypokalemic quadriparesis, sicca symptoms

## Abstract

Sjögren’s syndrome (SS) is an autoimmune disorder with glandular and extra glandular manifestations. The extra glandular manifestations include renal symptoms, primarily tubulointerstitial nephritis (TIN), while the glandular component involves the lymphocytic infiltration of exocrine glands. We describe the case of a 28-year-old woman who experienced two bouts of sub-acute onset recurrent flaccid quadriparesis in four months. Following the initial onset of quadriparesis, additional symptoms emerged, including dysphagia, dry mouth, and dry eyes, which raised the suspicion of underlying SS. Examination revealed a positive urine anion gap suggestive of distal renal tubular acidosis (DRTA), high urine pH, and severe hypokalemic hyperchloremic metabolic acidosis. The Schirmer test was positive after five minutes, and an additional workup confirmed positive anti-Ro and anti-La antibodies. A tissue biopsy collected from the sublingual salivary gland indicated lymphocytic infiltration, acinar atrophy, ductal dilatation, and epimyoepithelial cell islands on histological analysis, confirming SS to be the underlying cause of the symptoms. We stress in our conclusion that SS can also initially manifest without the classic symptoms of sicca and that it should be taken into consideration in cases of recurrent hypokalemic flaccid quadriparesis in the context of DRTA.

## Introduction

Sjögren’s syndrome (SS) is a rare autoimmune condition in which the immune system mistakenly targets and damages the body's glands and tissues, leading to chronic inflammation and dysfunction. It affects approximately 0.5% to 1% of the population, with a predilection for middle-aged women and a higher prevalence among Caucasians [1,2]. In SS, lymphocytic infiltration of the salivary and lacrimal glands causes reduced secretions manifesting as dry mouth and dry eyes. In addition to the exocrine glands, SS can affect the kidneys, lungs, nerves, and other organs. Sjögren’s syndrome is further classified into primary and secondary syndromes, where primary Sjögren's syndrome (pSS) can occur on its own or secondary Sjögren's syndrome (sSS) coexists with other autoimmune conditions, such as systemic lupus erythematosus and rheumatoid arthritis [2]. Nephrogenic diabetic insipidus, proximal tubular failure, and distal renal tubular acidosis (DRTA), the most prevalent form of renal involvement, can all be signs of tubulointerstitial nephritis (TIN). Renal tubular acidosis has been documented in 4.3% to 5% of patients with pSS [3]. The majority of patients with SS typically arrive with the regular symptoms first, followed by any related complications.

## Case presentation

A 28-year-old woman came with a complaint of paralysis in all four limbs for one day. The patient previously experienced identical symptoms four months prior and was treated with potassium supplements. In the two months after the initial episode of hypokalemia, she also experienced trouble swallowing food and gritty sensations in her eyes; she had been drinking water with every bite of food and using over-the-counter eye drops. The patient has no prior history of drug use, diabetes mellitus, hypertension, or hyperthyroidism. Her vital signs were within normal limits upon evaluation.

On examination, a deep, gasping respiration consistent with Kussmaul breathing was noted. The subsequent central nervous system (CNS) examination revealed no deep tendon reflexes and hypotonia (tone: 1+) in the muscles of the trunk, neck, and all four limbs (Table 1). Sensory and cranial nerve examinations were normal with intact bowel and bladder.

**Table 1:** The CNS motor examination showed hypotonia and absent DTR CNS: Central nervous system, DTR: Deep tendon reflexes

Clinical features	Findings
Strength	Decreased
Tone	1+ (hypotonia)
Deep tendon reflexes (DTR)	Absent
Babinski sign	Negative
Atrophy	Negative

The patient’s initial workup showed a normal complete blood picture (CBP), liver function test (LFT), and renal function test (RFT) values. Further laboratory investigations yielded the following results, which are summarized in Table 2.

**Table 2:** Laboratory investigations showed severe hypokalemic hyperchloremic metabolic acidosis, high urinary pH, and positive urine anion gap indicative of DRTA TTKG: Transtubular potassium gradient; ABG: Arterial blood gas; pCO2: Partial pressure of carbon dioxide; PO2: Partial pressure of oxygen; DRTA: Distal renal tubular acidosis

Investigation	Result	Reference range
Serum electrolytes		
Sodium	138 mmol/L	135-145 mmol/L
Potassium	2.1 mmol/L	3.5-5.0 mmol/L
Chloride	109 mmol/L	95-105 mmol/L
Bicarbonate	11.9 mmol/L	12-22 mmol/L
Calcium	9.4 mg/dL	8.5-10.2 mg/dL
Magnesium	2.10 mg/dL	1.82-2.30 mg/dL
Urine electrolytes		
Sodium	130 mmol/L	40-220 mmol/L
Potassium	17.23 mmol/L	25-125 mmol/L
Chloride	17 mmol/L	14-50 mmol/L
pH	8.2	4.5-7.8
Anion gap	10	<10 mEq/L
TTKG	>4	8-9
ABG		
pH	7.29	7.31-7.41
pCO2	21.7 mmHg	38-42 mmHg
pO2	98 mmHg	75-100 mmHg
Anti-Ro/SSA	138 AU/mL	0-40 AU/mL
Anti-La/SSB	142 AU/mL	0-40 AU/mL

**Figure 1:**
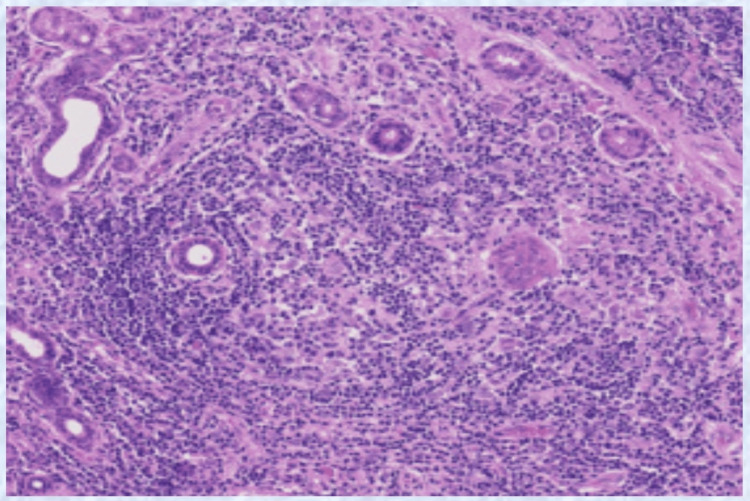
Histopathological examination of sublingual salivary gland showing lymphocytic infiltration (hematoxylin and eosin)

The ECG revealed hypokalemic changes, and the antinuclear antibody (ANA) profile was positive for Ro 52 and La 60 antigens. Histopathological analysis of the sublingual salivary gland revealed lymphocytic infiltration, acinar atrophy, ductal dilatation, and epimyoepithelial cell islands (Figure 1). Schirmer's I test was positive after five minutes; the ocular dye score was 6 according to the Van Bijsterveld scoring system.

Although false positive results can occur in a histopathological test while evaluating for SS, the factors affecting such results, like older age, history of trauma, and hepatitis C, were ruled out. The patient was diagnosed with pSS after meeting all the requirements in the revised international classification criteria, i.e., salivary gland involvement, histology, ocular signs, oral symptoms, ocular symptoms, and antibodies of either the la/SSB or ro/SSA antigens, or both [4].

The patient’s hypokalemia was treated with an intravenous potassium chloride infusion for two days at a rate of 20 mEq/hr. After two days, a repeat serum potassium test revealed 3.3 mEq/L, and the infusion was terminated. The patient was switched to oral syrup Potklor 10 mL/once daily. Pilocarpine eye drops were given for dry eyes, and the patient was advised to drink more fluids to treat the dry mouth. The patient showed significant progress and was discharged on day five with a three-month prescription of prednisolone 10 mg/once daily. At the three-month follow-up visit, the patient remained symptom-free with no evidence of hypokalemia or ongoing SS. Informed consent and signature were obtained from the patient regarding the use of her health information for the purpose of writing a case report publication.

## Discussion

We report a case of atypical Sjögren's disease that resulted in recurrent bouts of hypokalemic quadriparesis while concealing the conventional signs of the illness. After two episodes of hypokalemia, the sicca symptoms appeared, which prompted additional investigation into the underlying cause of SS. All common causes of weakness were ruled out, including peripheral neuropathy, polymyositis, myasthenia gravis, Guillain-Barre syndrome, and myopathy. The potential of paralysis caused by the toxin was ruled out because there was no history of snake bite or organophosphate (OP) compound exposure. A thorough workup for hypokalemia is essential in identifying atypical manifestations of SS. Normotensive hypokalemia was assessed as the patient is non-diabetic and non-hypertensive. Increased transtubular potassium gradient (TTKG) and potassium levels in the urine suggested potassium loss in the distal renal tubule, ruling out GI loss. The TTKG is a diagnostic tool employed to evaluate the renal response to potassium imbalances, helping to determine if the kidneys are adequately adapting to hyperkalemia or hypokalemia. In the context of hypokalemia, a TTKG value of less than 3 is considered normal; values greater than 3 imply inappropriate renal potassium loss, indicating renal potassium wasting. By combining signs of metabolic acidosis with distal renal tubule involvement, DRTA was identified.

Distal RTA is one of the extraglandular manifestations of SS. It is characterized as hyperchloremic, non-anion gap metabolic acidosis with impaired urinary acid excretion because of decreased hydrogen ion (H+) secretions by internalized cells in the distal nephron, which leads to decreased excretion of ammonium ion (NH4+) (H+). To preserve electroneutrality in the urine filtrate, there is an increase in distal potassium excretion [5]. Severe hypokalemia (<2.5 <2.5 mmol/L) leads to acute flaccid paralysis that can range from mild muscle weakness to severe paralysis [6].

In the present case, while the initial episode of hypokalemia was treated with potassium supplements, it ultimately led to recurrence. Meanwhile, SS persisted until the underlying cause was identified and addressed with prednisolone therapy, ultimately achieving resolution. The absence of characteristic sicca symptoms presented significant clinical challenges in assessing quadriparesis. The crucial connection between hypokalemia and DRTA ultimately proved pivotal in establishing an accurate diagnosis.

It is essential to consider Sjögren's disease in patients presenting with quadriparesis and recurrent hypokalemia with a subacute onset, as timely diagnosis and treatment can significantly impact patient outcomes and prevent complications. Notably, middle-aged women with severe hypokalemia, even in the absence of typical glandular symptoms, should be evaluated for SS to ensure prompt identification and management of this potentially debilitating condition.

## Conclusions

This case underscores the importance of considering Sjögren’s syndrome as a potential underlying cause in patients presenting with recurrent hypokalemic quadriparesis, especially in middle-aged women, even in the absence of typical sicca symptoms. Early recognition and treatment of Sjögren’s syndrome can prevent complications and improve patient outcomes.

Our patient's symptoms were successfully managed with appropriate potassium supplementation and immunosuppressive therapy, highlighting the necessity of a thorough diagnostic workup for unexplained hypokalemia. This case thus serves as a reminder to clinicians to maintain a high index of suspicion for autoimmune disorders in atypical presentations, ensuring timely diagnosis and effective treatment.
